# Machine learning predictions surpass individual mRNAs as a proxy of single-cell protein expression

**DOI:** 10.1186/s13059-026-04083-1

**Published:** 2026-04-22

**Authors:** Josephine Fisher, Oliver Wood, Samuel Bullers, Lynne Murray, Li Li, Matthew A. Jackson-Wood

**Affiliations:** 1 Gilead Sciences, 9400 Oxford Business Park, Garsington, Oxford, OX4 2HN UK; 2 Gilead Sciences, 333 Lakeside Dr, Foster City, CA 94404 USA

**Keywords:** Machine, Learning, Protein, Prediction, Transcriptomics, Proteomics, Single-cell, CITE-seq

## Abstract

**Background:**

Expansive repositories of scRNA-seq data are now available. These are often analysed assuming that mRNA abundance reflects expression of the cognate protein. However, post-transcriptional/translational regulation and the sparsity of measurements in single-cell data make mRNA an inadequate proxy for protein. Methods to quantify surface proteins alongside scRNA-seq exist but are less widely adopted. Machine learning approaches for protein imputation from scRNA-seq data have been published, which learn transcriptome-wide patterns that predict protein expression where data for both is available. These models can then be applied to infer surface protein expression on scRNA-seq only data sets, increasing their utility.

**Results:**

We test 9 machine learning methods for predicting single-cell protein expression, comparing the accuracy between methods and compared to using cognate mRNAs alone. Overall, machine learning -based protein predictions across methods outperform direct inference from mRNAs, including cases where proteins absent by mRNA are successfully predicted by the wider transcriptome. When comparing models trained on restricted cell types and across different datasets/tissues, we find that the overlap in cell type composition of training and test data is an important determinant of prediction accuracy. We also compare computational resource requirements to guide method selection.

**Conclusions:**

These results reiterate that single-cell mRNA abundance is not a reliable proxy of cognate protein expression and that whole-transcriptome based imputations can improve upon them given appropriately trained models. However, limitations to the generalisability of these methods persist, notably a requirement for highly similar training data, which may limit the current scope of applications.

**Supplementary Information:**

The online version contains supplementary material available at 10.1186/s13059-026-04083-1.

## Background

There has been widespread adoption of methods to profile transcriptomics at the single-cell level (scRNAseq). This has resulted in an abundance of published scRNAseq datasets, including several high-profile projects to create atlases of human health and disease [1–4]. While scRNAseq is a powerful tool, it should be used with respect to its limitations. Limited read depth, transcript bias, and low capture efficiency can produce highly sparse scRNAseq data, with lower abundance genes often failing to be detected [5]. This, alongside the complexity of post-transcriptional and translational regulation, means that transcript abundance may not reflect functional protein levels.

New technologies seek to expand options for single cell profiling to measure multiple modalities simultaneously, some of which quantify protein expression alongside mRNA. These include CITE-seq strategies, such as AbSeq from BD Bioscience and Total-Seq from Biolegend, which use oligonucleotide barcoded antibodies to label potentially 100 s of cell surface proteins per experiment [6–10]. Subsequently the cell is lysed, and protein abundance measured alongside the mRNAs by sequencing. Such data presents an opportunity to quantify the relationship between mRNA transcript and surface protein expression. However, the relative novelty of these approaches and challenge of generating and profiling oligo conjugated antibody panels means that uptake remains comparatively limited. As an alternative to direct measurement, a multimodal reference may be used to train a machine learning (ML) model and impute protein levels on the large volume of scRNA-seq data already available.

Various methods have recently been published to infer protein levels from scRNAseq using models trained on a multimodal reference. The package Seurat, commonly used for single-cell data processing, is capable of graph-based reference mapping to assign protein expression based upon cell–cell distances [11]; sciPENN, totalVI, BABEL, scMMT and cTPnet all apply some variation of a neural network to generate predicted protein expression values [12–15]; foundation models such as scTranslator expand upon these deep learning approaches by pretraining a model on a broad knowledge base [16]; the method scLinear uses a comparatively simple approach, applying dimensional reduction followed by linear regression [17]; an unsupervised approach, SPECK, uses reduced rank reconstruction for protein prediction directly from RNA expression alone, clustering each gene separately [18]. There is outstanding need for a comprehensive, independent assessment of these advanced methods, and their value over using mRNA abundance alone as an estimator. Additionally, inter-method comparisons of performance when tested on different features and tissues are lacking. To address this, we present a comprehensive evaluation of 9 prediction methods, seeking to define their strengths and limitations.

Protein expression predictions were made on PBMC CITE-seq data from healthy donors, split into training and test partitions [11]. We measured correlation between predicted and measured expression values and compared between methods and against the performance of the cognate mRNA as a proxy. We also evaluated the main factors driving predictive power by training and testing methods on cell type restricted versions of the PBMC data where applicable, and also within and between a wider selection of CITE-seq datasets covering a range of tissues and disease states. Overall, we found transcriptome-wide predictions of cell surface protein expression outperformed using mRNA in isolation for most methods. Baseline expression level and variability between cell types were major determinants of a protein’s predictability. When comparing across tissues/datasets, we found an overlap of cell type composition between test and training data is needed for accurate predictions. These findings highlight that mRNA can be a poor representative of cell surface protein abundance and that there is sufficient information in the wider transcriptome to make a more accurate inference of protein expression. However, current methods require a well-matched multi-modal reference, which may limit their applicability.

## Results

### Gene mRNA abundances are an inconsistent proxy of surface protein expression

**Fig. 1:**
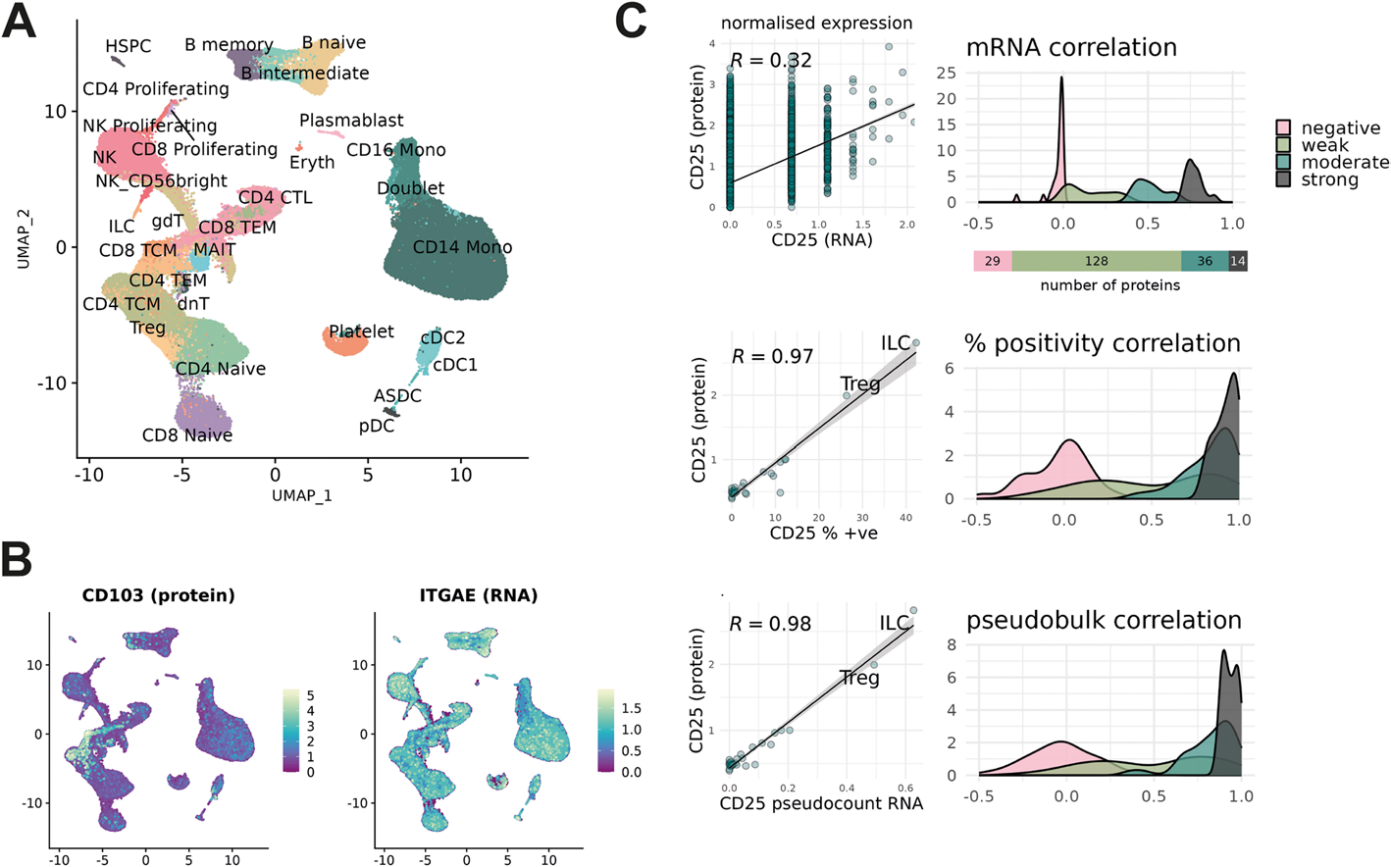
RNA–protein correlation in the Hao et al. CITE-seq data. **A** UMAP showing the immune cell types profiled in the Hao et al. PBMC CITE-seq data. **B** CD103 expression measured by normalised protein and normalised RNA. **C** Density plots showing Pearson correlation of RNA–protein pairs measured by normalised RNA, proportion of RNA positive cells, and by RNA pseudobulk count. Features binned into correlation brackets according to correlation between normalised mRNA expression and measured protein

We first measured the naïve correlation between proteins and their mRNA abundances as a baseline expectation of correspondence. For this we utilised a large CITE-seq dataset profiling PBMCs from healthy volunteers from a vaccine trial, published by Hao et al. [11] (Fig. 1A). This has been used extensively in demonstrations of multimodal single cell analyses [19–23]. It is comprised of 161,764 cells, classified into 31 functional types.

Of the 228 proteins measured, 207 could be directly aligned with a single gene measured in the RNA data (excluding non-specific and control antibodies). We computed Pearson correlation between the measured protein and its corresponding mRNA (Additional file 1: Fig. S1). Several cases were identified in which the expected expression profile as estimated by RNA was dramatically different from the expression observed by antibody measurement. A pronounced example was the tissue-residency marker CD103, showing extracellular protein detected only in a narrow subset of T-cells despite uniform abundance of RNA counts across all immune cell types (Fig. 1B). Of the 207 protein-mRNA pairs, 14 proteins had strong [0.7,1] Pearson correlation with normalised RNA abundance, 36 had moderate [0.4,0.7) correlation, 128 had weak [0,0.4) correlation, and 29 had negative correlation (Fig. 1C).

Proteins that were well approximated by RNA include several canonical immunophenotyping markers, such as CD3, CD16, CD14 and CD20, but some markers of lymphocyte activation and cell state, such as CD25, showed poor correlation with RNA in circulating PBMCs (Fig. 1C). Some of these cases align with known biology; isoforms such as CD45RA and CD45RO are heavily controlled by post-transcriptional processes such as alternative splicing, so aren’t expected to align well with PTPRC expression [24]. Other discrepancies have less obvious causes, and our results suggest a low probability that any given gene will have RNA measurements well-correlated with protein expression. To address this difficulty we explored alternative metrics by which RNA can be used as an estimator for extracellular protein, computing either the percentage of RNA positive cells or the pseudobulk count over a cell type (Fig. 1C). Both approaches could recover a well-correlated, population-level estimate of median protein expression, generating similar results between the two methods. Examining changes in group distributions, we found these metrics to improve correlation values for strong and moderately correlated RNA–protein pairs. However, they largely failed to produce high correlation values for proteins with negative or low-valued correlation with the naïve mRNA proxy. Additionally, these metrics cannot be used where single-cell context is important. There remained a set of 112 proteins that failed to achieve correlation ≥ 0.7 using approximation by RNA counts, percentage positivity, or pseudobulk count (Additional file 1: Fig. S1, Additional file 2: Table S1). CD103 is one such case, remaining resistant to these approaches due to fundamental disagreement between RNA and protein measurements.

### Whole transcriptome gene expression predicts protein abundance better than source mRNA alone

Motivated by the insufficiency of protein estimation directly from source mRNA, we evaluated 9 published approaches for predicting protein abundances using full whole-transcriptome profiles. Performance was tested on the Hao et al. CITE-seq dataset [11]. We took a randomly sampled 20% fraction as the test (i.e. query) data and trained each method on the remaining 80%. For scTranslator few-shot training, 1000 cells from the test fraction were used for fine tuning. Pearson correlation between predicted and measured protein expression was used as the primary evaluation metric, henceforth referred to as the prediction correlation. Normalisation of measured expression was conducted separately for each method, to match the expected pre-processing format.

**Fig. 2:**
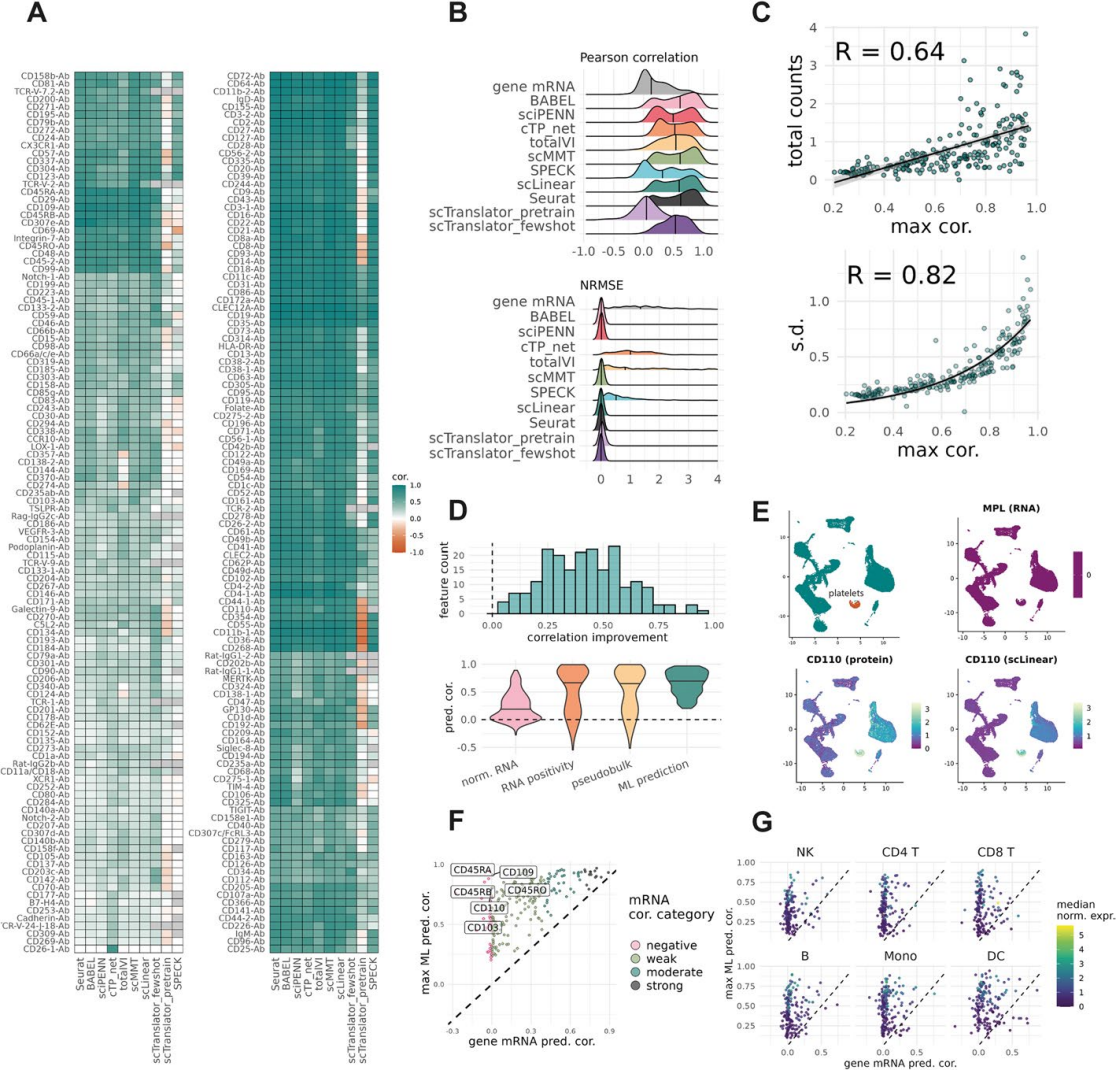
Improvement in prediction correlation using ML methods. **A** Heatmap showing Pearson correlation between predicted and measured protein expression values. Grey boxes indicate the protein was not imputed. **B** Density plots showing distribution of prediction correlation and NRMSE for each method. **C** Scatter plots showing maximum prediction correlation against total protein counts and standard deviation in the training data. **D** Histogram of difference in correlation coefficient between maximum prediction correlation and gene mRNA correlation. **E **UMAP panels highlighting platelet population and showing MPL (CD110) RNA expression against normalised protein expression and scLinear predicted values. **F** Gene mRNA correlation plotted against maximum prediction correlation across methods, coloured by correlation categories defined in Fig. 1C. Features with large correlation improvement are annotated. **G** Correlation scatter as in **F**, split by cell type and coloured by CLR normalised expression of the protein

For many proteins there was low variability in prediction correlation across the different approaches, indicating a uniform ability across methods to impute expression (Fig. 2A). A notable exception is the unsupervised strategy SPECK, which does not utilise protein data in its training. SPECK consistently produced predictions with lower correlation than most other methods. In several cases SPECK produced near-zero prediction correlation for features which were well-predicted by other methods, such as CD324 and CD68 (Fig. 2A). SPECK also failed to produce a prediction for some features owing to a lack of RNA detection in the training data, likely due to dropout. scTranslator similarly suffers from an inability to predict proteins whose antibody cannot cleanly be aligned to a gene symbol, due to tokenization restrictions. Predictions generated by scTranslator few-shot training generally produced high correlations, on par with other methods. When assessing correlations non-parametrically, scTranslator few-shot was the best method suggesting that predicted values are better used as ranks than as proxies for abundance (Additional file 1: Fig. S3B). However, the pretrained model with no finetuning performed poorly by all metrics. Most methods showed bimodality in prediction correlation across features, indicating a discrete set of features that are more challenging to impute than others (Fig. 2B). We sought to identify what characteristics might make a protein easier or more difficult to impute and found a linear association between the maximum prediction correlation and total number of counts of a protein (Fig. 2C, Additional file 1: Fig. S3C). Proteins with high standard deviation also trend towards having better average prediction correlation. Thus, features with strong and distinct signal in the data appear easier for prediction methods to impute.

Across all tested methods there were many proteins that consistently showed improved prediction correlation with genome wide imputation compared to direct estimation from cognate mRNA (Fig. 2D). At worst, ML imputation maintains comparable maximum prediction correlation to aggregate RNA metrics and produces no negative correlation values. Of the 207 proteins for which cognate RNA data is available, 79 fail to achieve moderate prediction correlation > 0.4 by either direct RNA estimation or aggregate RNA measures (Additional file 2: Table S1) but 57 of these achieve maximum prediction correlation > 0.4 when using the best ML method.

The utility of protein imputation is highlighted by the feature CD110, a thrombopoietin receptor commonly detected on platelets [25]. Platelets contain no nucleus, and typically have only a small amount of legacy mRNA transferred from megakaryocytes [26]. The CD110 transcript MPL is undetected in the Hao et al. test data rendering direct estimation impossible, however CD110 protein is enriched on the platelet surface. Using the full landscape of platelet RNA, ML prediction methods can learn the transcriptional signature of platelets and recover CD110 enrichment in the population (Fig. 2E). As a result, average prediction correlation across methods for CD110 is strong, at 0.75. Other examples benefiting from imputation include protein isoforms, which show a marked improvement in correlation using ML methods. Both CD45RA and CD45RO are predicted with much greater accuracy by ML methods than by estimation from PTPRC mRNA alone (Fig. 2F). Proteins that are well correlated with mRNA abundance such as CD20 and CD16 remain accurately predicted by ML methods, with some also showing a slight improvement in correlation. Splitting the data by cell type there were a small number of features that were better represented by mRNA than predictions. But for the majority of features, predictions improved upon mRNA in all cell types (Fig. 2G).

For all features, the maximum prediction correlation over ML methods was higher than that of gene mRNA alone. A subset of 22 proteins were poorly estimated by both normalised RNA-proxy and ML methods, having prediction correlation < 0.4 in both cases (Additional file 2: Table S1). These weaker predictions may be due to low abundance or low variability of the proteins in the training data. Generally, our results show ML imputation to either match or improve upon the prediction correlation produced by normalised mRNA expression.

### Prediction accuracy depends strongly upon transcriptional signatures of cell type

**Fig. 3:**
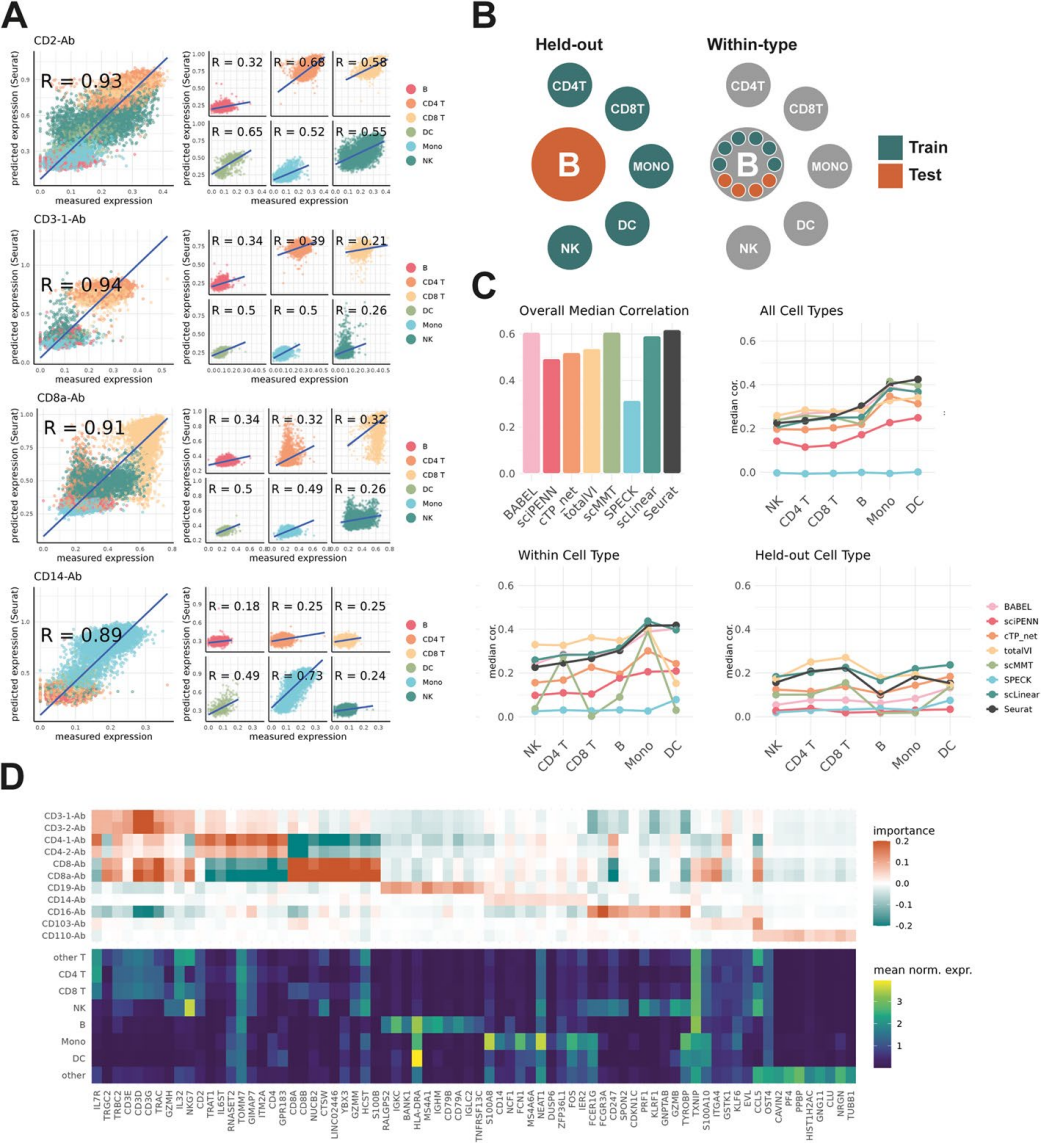
Sensitivity of prediction to cell type. **A** Scatter plot showing prediction correlation of examples features from Seurat, computed over all cells and over cell-subtypes. **B** Schematic of training and test sets in investigation of cell type sensitivity. **C** Panels showing bar plot of overall prediction correlation across all cells, alongside line plots of median prediction correlation in each cell type individually (all cell types), when only one cell type is used for both testing and training (within cell type), and when a cell type is held out as the test set (held-out cell type). **D** Heatmap of top 10 most important RNA features in the scLinear prediction model for predictions in Fig. 2A, shown for a selection of proteins

Given that the proteins best predicted were often the most variable across the dataset we investigated if cell type, a likely prominent source of variation, was an influential factor in ML inference. We found several cases where within-cell type prediction correlation was poor despite high overall prediction correlation across all cells (Fig. 3A). To interrogate this further, we ran protein imputation with 8 methods on two variations of the Hao et al. data (Fig. 3B). In the first, each cell type was isolated and used for both training and test partitions as before. This was intended to explore changes in performance with reduced phenotypic variability in the training data. In the second, one cell type was withheld as the test set and prediction methods were trained on the remaining cells. This tested the methods ability to infer protein expression on unseen cell types. We refer to the two training variations as within-cell type and held-out cell type, respectively. We compared them to the all-cell case, where the entire data was sampled to produce testing and training fractions (Fig. 3C). Similar testing of scTranslator was not performed as all cell types are captured in the pretraining of the model.

In the within-cell type case we saw mostly similar prediction correlations as in the all-cells case, with only minor reductions in median prediction correlation for most methods. However, scMMT showed pronounced sensitivity to the restriction of cell type information, producing substantially lower prediction correlation in NK cells, CD8 T cells, B cells and dendritic cells. In the held-out tests all methods exhibited uniform decline in prediction correlation, with no methods achieving median prediction correlation over all features greater than 0.3. This demonstrated sensitivity of reference-based prediction to the cell type composition of the training data.

We further explored the cell type dependency by examining feature importance in model predictions. Given the black-box nature of most ML methods, we focused on scLinear which inherently provides feature importances. We extracted the top 10 most important mRNA features for predicting a selection of known cell type marker proteins (Fig. 3D). These top features were predominated by cell type markers. For instance, protein expression of the B-cell marker CD19 was primarily determined by mRNA expression of other canonical B-cells markers such as MS4A1 and CD79. The T-cell markers CD3, CD4 and CD8 were strongly informed by their corresponding mRNAs and other genes primarily expressed in T-cells. We did also observe a small number of non-obvious genes with high importance for protein imputation but also broad, cross-phenotype, expression. This suggests more complex interactions with other genes in the model providing predictive information. We further found feature importance to align with expected biology. For example, protein expression of the tissue residency marker CD103 is informed by a collection of mRNA features with related function: the genes EVL and ITGA4 both have established relevance to immune tissue infiltration [27, 28], and CCL5 is understood to recruit peripheral immune cells to inflammatory sites [29, 30].

### Dissimilar training data reduces prediction performance

**Fig. 4:**
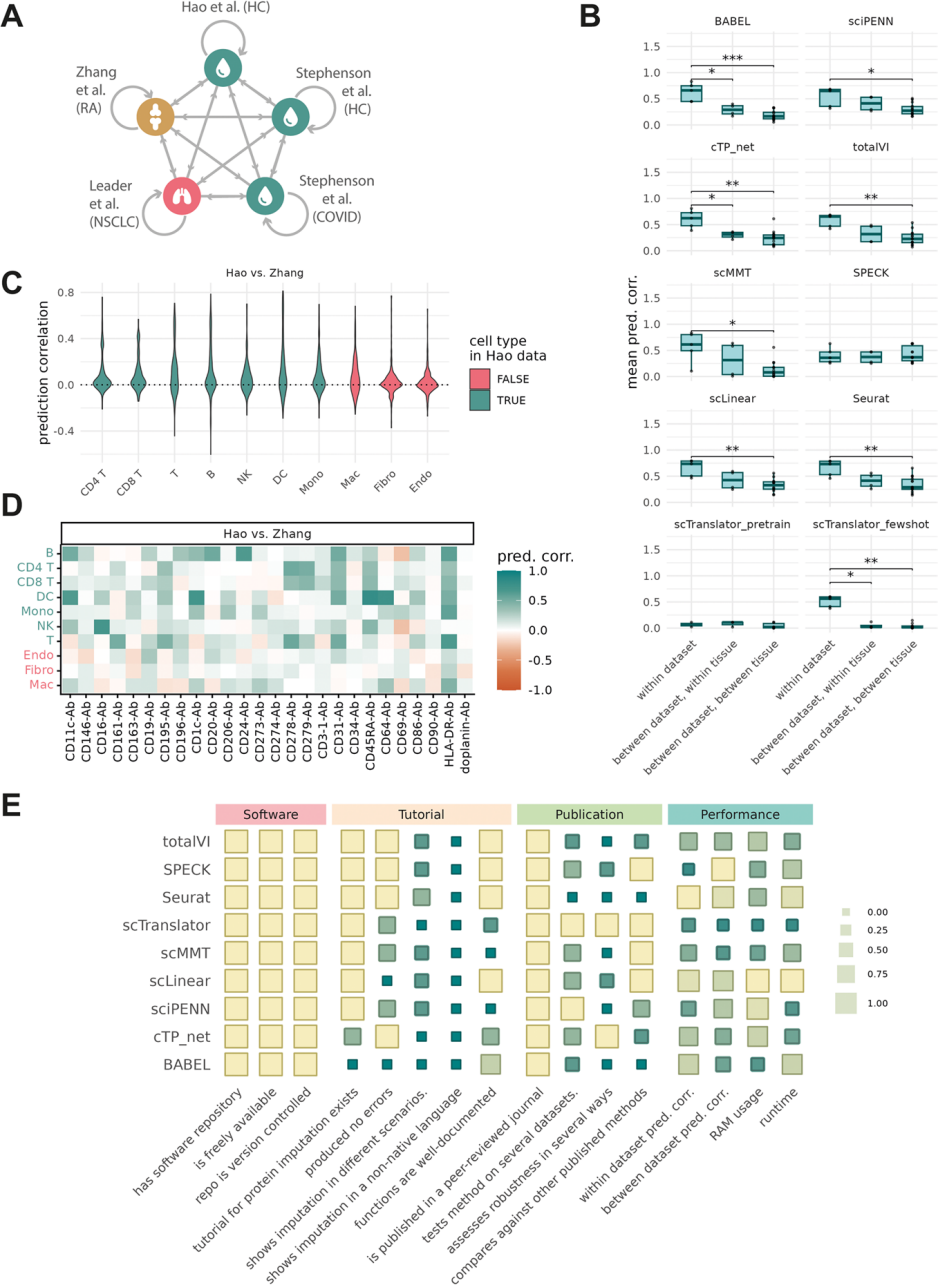
Volatility in cross-dataset prediction. **A** Schematic of dataset-to-dataset training and testing operations. **B** Boxplots of median prediction correlation over proteins for tests within dataset, between datasets of the same tissue, and between datasets of different tissue. Cross-disease predictions for Stephenson et al. were omitted. Stars indicate exceeded thresholds on Bonferroni adjusted *p*-value from Wilcoxon tests with * = 0.05, ** = 0.01, *** = 0.001. **C** Violin plot of Seurat-derived prediction correlation values for each antibody shared between datasets, with Hao et al. as the training set and Zhang et al. as the test set. Correlations are computed for each cell type individually. **D** Seurat-derived prediction correlation values as in C) shown for each protein individually. **E** Summary table of method usability, showing score in range [0, 1]  for each criteria with 1 indicating the best scoring method(s). scTranslator evaluation is for few-shot prediction only

Given the observed importance of cell type similarity on prediction accuracy within a single dataset, we aimed to explore the capabilities of protein prediction methods when training and testing across datasets from similar or diverse tissues. We included an additional PBMC CITE-seq dataset from Stephenson et al. comprised of both healthy and COVID samples, a non-small cell lung cancer (NSCLC) dataset from Leader et al., and synovial samples from rheumatoid arthritis patients profiled by Zhang et al. [31–33]. To ensure fair comparisons, we generated distinct 20,000 cell test and train datasets from each study (and from Hao et al.) and performed all pairwise predictions (Fig. 4A). Prediction correlations to measure protein was assessed for the 26 proteins which shared the same antibody clone across all datasets.

The highest prediction correlations were observed in the within-dataset comparisons (Fig. 4B, Additional file 1: Fig. S4). For all trained methods, we saw a statistically significant drop in prediction correlation between different tissues, and slightly better performance between datasets within a tissue. Few-shot scTranslator prediction was only successful when trained on cells from the same experiment, producing very poor prediction correlation otherwise. We also noted scMMT performance dropped when using the 20,000-cell subset of Hao et al. data compared to prior results. Exploring more deeply, we found performance improved when training reached a 50% (~ 80 K cells) fraction of the data suggesting this method may be more sensitive to sample size (Additional file 1: Fig. S5A). The Leader et al. lung data proved consistently poor as a training reference (Additional file 1: Fig. S4). On closer inspection we found this may be due to the quality of the CITE-seq readouts in the data where several well-characterised cell type markers (e.g. CD3, CD4, CD19) had indistinct expression patterns (Additional file 1: Fig. S6A). Conversely, predictions of these markers on the Leader data using the Hao et al. PBMC data as a reference generated the expected expression profiles (Additional file 1: Fig. S6B).

Focusing on the Hao et al. PBMC vs Zhang et al. synovial comparison, we sought to explore if missing cell types contributed to the drop in prediction performance. Across all the proteins considered, predictions tended to be more correlated when predicting on cell types present in both datasets (Fig. 4C). Exploring individual proteins within the Hao-Zhang comparison (using the Seurat predictions as an example) we can see specific examples such as the endothelial and fibroblast markers CD31 and CD90 (Fig. 4D), which are poorly predicted for those cell types as neither is present in the Hao et al. reference (Additional file 1: Fig. S7). Prediction correlation is improved for some cell type markers where similar cell types are seen in both datasets, such as CD20 on B-cells and CD11c on dendritic cells. However, this is not uniformly the case: T-cells are common to both datasets but CD3 maintains low prediction correlation across cell types. Overall, cross-tissue prediction produced more variable outcomes than within tissue predictions.

### Comparison of method usability

As we observed similar performance between methods, we also compared their usability. We ranked methods by computational requirements, software documentation, thoroughness of the associated publication, and prediction accuracy using an adapted version of a framework used in benchmarking single-cell integration and trajectory inference methods [34, 35]. Methods were ranked relative to one another, with 1 indicating the best (Fig. 4E). There was substantial variance in the quality and detail of tutorials provided by software authors. Prediction accuracy was ranked between and within datasets and memory and execution time were ranked according to the average over pairwise dataset comparisons. Overall, scLinear and Seurat emerged as prominent fast-running methods with reasonable resource requirements and strong prediction performance.

## Discussion

Our analyses confirm that RNA does not always provide an accurate proxy measure for extracellular protein. Many post-transcriptional and post-translational mechanisms could explain the mismatch between intracellular RNA, and protein at the cell surface. Alternative splicing, trafficking apparatus, protein–protein interactions and decay/secretion rates all influence the quantity of protein maintained on the cell surface [36]. These complicating factors alongside the data sparsity seen due to the low depth of scRNA-seq make it difficult to determine how a protein relates to its corresponding RNA. To combat the latter, one can instead use an aggregate measure over a population of interest. We found that RNA positivity and pseudobulk expression both produce very similar outcomes and often provide estimates well-correlated with median protein expression in the target population. Unfortunately, these summary metrics remove the single-cell granularity of data and still perform poorly where there is a biological disconnect between mRNA and protein, as with protein isoforms. Our analyses suggest that whole transcriptome-based prediction provides a viable alternative to protein estimation and can recover obscured RNA–protein relationships through an understanding of cellular phenotype. Caveats remain regarding the selection of suitable training data and poor performance on a limited portion of protein features.

Of the 9 tested methods, all produced predictions well-correlated with measurements for a subset of core features with values consistently better than mRNA alone. Methods did show variable performance over the full set of proteins, with SPECK in particular struggling in cases where there was no capture of cognate RNA. Clearer differentiating factors between methods were runtime, resource usage, and sensitivity to scale and composition of training data. scLinear and Seurat both maintained performance stable to change in size of the training data, and both methods complete quickly without high computational resource requirements. Of the available options, we recommend these methods as accessible and capable strategies for protein imputation.

Prediction correlations varied strongly across proteins, with individual features having uniformly strong or weak correlations over methods. This suggested some inherent qualities of proteins may cause to them be challenging to accurately impute. Our results suggest that ML-prediction is not a one-size-fits-all solution for any target. We found that well-predicted proteins tend to have high counts and variability in the data, generally having a strongly discriminated pattern of expression across cell types. Methods tended to maintain good performance on cell type defining features already well-approximated by RNA expression, such as CD3 and CD4. They could further produce modest improvement in prediction correlation in many other cases with poorer RNA correlation, and could rescue intractable features for which RNA is entirely undetected. However, some key immunoregulatory proteins remain poorly represented by both cognate mRNA and prediction methods. The checkpoint receptors BTLA (CD272), TIGIT and CTLA4 all failed to achieve strong average prediction correlation (> 0.7) by ML prediction on the Hao et al. data, but did pass that threshold by RNA aggregation metrics (positivity, pseudobulk) (Additional file 2: Table S1). In such cases we found no reliable single-cell resolution estimation strategy.

We saw reduced performance when training and testing on different cell type subsets of the PBMC data, and when training and testing between datasets of varied disease and tissue origin. This implicates dataset-specific cell type transcriptional signatures as strong determinants of model performance. Feature importance in scLinear prediction supported this, revealing the genes with most predictive power to have highly differentiated patterns of expression by cell type. In some cases, low between-dataset performance might be attributable to poor quality protein quantification creating indistinct cell type signatures and ultimately noisy training data. In others, differences in the cell type composition of the samples appears to render the training data incapable of accurately recapitulating expression on unseen cell types. Whether this dissimilarity between cell type signatures is specifically driven by tissue or disease of the samples remains unclear. Reductions in performance may be explained by overfitting to the compositional profile of the reference data, or by the explanatory transcriptional features of seen and unseen cell types not having enough in common to accurately predict protein expression. These outcomes suggest a need for high quality training data of a similar phenotypic composition to the test use case data. This may limit applicability of these methods where similar CITE-seq data are not available. Even in such instances it may be possible to design efficient, cost-effective hybrid studies. One could apply CITE-seq to a small subset of samples and then use ML-imputation to infer protein on higher-throughput, highly multiplexed RNA data that can be generated more affordably [37–39].

New emerging computational methods may be able to overcome some of these challenges. Foundation model strategies seek to create a highly generalisable framework that can perform well across a range of prediction tasks. However, we found the only foundation model currently specialised for this task (scTranslator) to produce poor outcomes in the absence of fine-tuning using cells from the same experiment as the query, despite pretraining on a large and diverse body of data. This is in line with the authors original results where few-shot training was used in most comparisons [16]. The limited performance of scTranslator without fine tuning suggests there is work to be done before consistently accurate zero-shot prediction can be achieved. The relatively small number of cells required for few-shot optimisation may be beneficial in certain situations where limited training data is available. But the poor performance of scTranslator without fine tuning negates the supposed benefit of foundation approaches over the more traditional ML methods that require matched training data. Our results stand alongside recent evidence showing the current generation of single-cell foundation models in other applications may not improve upon more traditional statistical approaches in other applications where they have been tested [40, 41].

Experimental advances may also provide another source of innovation in this space. There are experimental challenges with current CITE-seq methods: panels are limited to measuring ~ 100–200 cell surface proteins in a single experiment, and the requirement for validated antibodies limits the ability to explore less studied or novel proteins. New CITE-seq protocols are enabling the measurement of intracellular protein and post-transcriptional modifications, expanding capabilities beyond cell surface proteins alone [42–44]. Recent advancements in sample preparation and equipment sensitivity have been used to apply mass spectrometry for untargeted single-cell proteomics, albeit currently very limited in throughput and sensitivity [45]. This increasing range of experimental and bioinformatic tools to study protein expression will further reduce the reliance on transcriptomic data as a proxy and could provide new sources of training data for inference models.

## Conclusions

Our study highlights the inadequacy of mRNA abundance as a proxy of protein expression in single-cell data. We have evaluated the utility of transcriptome-wide ML based protein prediction as an alternative approach and define the limitations of these methods. Accurate estimation of cell surface protein expression is of great importance for the definition of cell types, study of disease pathology, and identification of therapeutic targets. Thus, adoption of models to estimate cell surface protein expression more accurately could prove valuable to improve the utility of large existing repositories of scRNA-seq data. Focus should be given to identifying and generating suitable reference datasets to learn from and the development of more advanced, globally applicable, modelling approaches to predict protein measures from single-cell transcriptomes.

## Methods

### Data and preprocessing

We explored performance of protein imputation methods on four CITE-seq datasets. In all cases we used log-normalized RNA counts, and normalised protein according to expected format for the imputation method. In our comparisons between datasets we retained only those antibodies with the same clone across experiments.

The first dataset is the Hao et al. PBMC data (GSE164378) [46]. This dataset describes blood samples obtained from eight volunteers enrolled in an HIV vaccine trial, taken at baseline and at days 3 and 7 following vaccine administration. Whole transcriptome RNA profiles were measured on 161,764 cells. A panel of 228 antibodies was used to profile surface proteins on all cells. This data was downloaded as a Seurat object provided by the New York Genome Center, used with no further processing or cell filtering. The second CITE-seq dataset used is the non-small cell lung cancer data presented by Leader et al. RNA counts are provided under accession GSE154826 and protein data is available via the github repository [47, 48]. The data contains RNA profiles for 361,929 cells, taken from tumour and non-involved human lung tissue. Of these, protein was measured on a subset of 230,820 cells with varied panels. For correlation analyses, only cells with protein measurement were included. The third dataset is a CITE-seq profile of healthy and Covid blood samples presented by Stephenson et al. under accession E-MTAB-10026 [49]. The data contains 97,039 healthy cells and 527,286 Covid cells, which were treated as distinct sets for our analyses. The final dataset is rheumatoid arthritis synovial CITE-seq data presented by Zhang et al. and available under Synapse accession syn26710600 [50].

### Aggregate RNA metrics

In our evaluation of correspondence between RNA and protein, we explored the value of using percent positivity and pseudocount estimators. Percent positivity was computed simply as the proportion of cells in a group in which the transcript was detected. Pseudobulk counts were computed using the Seurat function *AggregateExpession().* RNA counts were summed over donor and cell type. The final pseudobulk value per cell type was taken as the mean value over donors.

### Protein imputation

We ran 9 protein prediction methods using a custom Nextflow pipeline [51, 52]. Code for model training and prediction was sourced from author-provided github repositories or vignettes for each method. Arguments controlling prediction strategy were set to default according to vignettes or example code. Prior to prediction the data was partitioned into a training and test set, taking an 80:20 split and selecting cells at random with respect to preservation of cell type proportions. Predictions methods were trained and tested on the same partitions. Predictions made on the test data were evaluated against the measured protein by computation of Pearson correlation and NRMSE. All imputation methods were set to apply built-in preprocessing of RNA and protein data, as defined in the published software.

### Evaluating predictions

When evaluating baseline correlation between mRNA and protein, we first had to determine suitable alignment between RNA and protein features. Where antibodies had no sensible, direct mRNA equivalent (isotype controls, TCR binding antibodies) they were dropped from correlation analysis. Where correlation was not possible to evaluate due to high sparsity and RNA dropout, we set prediction value to zero to facilitate comparison with other methods. RNA–protein correlations were computed only on cells from the test set, for parity with ML-prediction correlations. Where prediction correlation was evaluated over a set of features, we took the median correlation value. Where prediction correlation was evaluated over ML methods, we took the maximum correlation value. Where root mean squared error (RMSE) is used, it is divided by the standard deviation of the measured protein expression to produce the normalised RMSE (NRMSE).

Each method conducts or expects distinct preprocessing of protein counts prior to training and computation of evaluation metrics. For fair comparison we normalised ground truth protein count values separately for each method, according to the comparative strategy and preprocessing described by method authors. Seurat, BABEL, SPECK, scMMT and scLinear predictions were compared against CLR normalised counts. totalVI predictions were compared against scanpy log1p transformed counts. For sciPENN, cells were first normalised by counts-per-cell. Following this, values were log1p transformed and z-scored by gene within donors. cTPnet uses an abundance transformation described by Stoeckius et al. [9], and scTranslator applies scaled max–min normalisation. These were respectively applied to the raw counts before metric evaluation for both methods.

### Prediction benchmarking

To assess comparative performance of prediction methods Nextflow configuration was used to ensure each prediction process had access to identical memory and CPU resources. The trace and execution reports generated by Nextflow were used to report runtime and resource usage for each method.

To explore how prediction results change with differences in training data, we ran the methods under four separate variations.

1. The Hao et al. data was subsampled into 6.125%, 12.5%, 25%, 50% and 100% fractions. Following this, it was split into a training and test set in an 80:20 ratio. Prediction methods were then trained and tested. This approach was intended to measure differences in performance as the size of the data increases.
2. For each cell type group in the set B, CD4 T, CD8 T, NK, Mono and DC the selected type was entirely held out of the Hao et al. data as the test set. Methods were trained on the remaining cells, and prediction correlation measured on the held-out cells. This approach is intended to assess ability to accurate predict protein on unseen cell types.
3. For each cell type group in the set B, CD4 T, CD8 T, NK, Mono and DC the selected type was taken from the Hao et al. data and treated as the entire data. It was split into a training and test set in an 80:20 ratio. All other cells were discounted. Prediction methods were trained and tested on only one cell type at a time. This method was intended to measure capacity to detect within-cell type variation in protein expression.
4. For each pairwise combination of CITE-seq datasets, methods were trained on one dataset and tested on another. Each testing and training set consisted of a 20 K cell subsample of the full data. This approach was intended to measure the influence of experimental quality, variable data composition and tissue effects on method performance.

The few-shot scTranslator approach is applied consistently using 1000 cells, without respect to variable size of the overall training fraction. Further, the scTranslator pretrained model has built-in knowledge from a broad spectrum of cell types, rendering it unsuited to testing the influence of cell type composition. We therefore excluded scTranslator from benchmarking analyses 1–3, and ran scTranslator with different resource specification to other methods to facilitate efficient training.

### Feature importance in scLinear

While not possible for all prediction methods, we were able to extract a measure of feature importance from the linear models generated by scLinear. As described by the package authors, feature importance is calculated as the Jacobian matrix of the predicted ADT values, with respect to the input RNA matrix. This was computed as the product of the Jacobians for the singular value decomposition, linear regression and z-score normalisation components of the model. Further details of computation are given by Hanhart et al. [17]. Genes with large, positive importance value are understood to be influential over prediction outcomes.

### Usability scoring

We measured usability with an adapted version of a framework previously applied to benchmark methods for single-cell integration and pseudotime trajectory inference [34, 35]. Methods were evaluated according to 16 criteria (Additional file 3: Table S2) which measure both how accessible and accurate the software is, and how thoroughly the publication evaluates the method against other approaches. The first three criteria simply check that the method be available in a version-controlled repository, and that the underlying code is freely available and executable on an open-source platform. Five conditions are used to evaluate the quality of available software tutorials. These are that a tutorial exists, be well-documented, that it shows the imputation method in different scenarios, that it shows how to run the method in a non-native language, and that running the tutorial produces no notable errors. A set of four criteria evaluate the publication. The first is simply that the method is published in a peer reviewed journal. The others assess the number of datasets on which the method is tested, the diversity of robustness assessments, and breadth of comparison with other protein imputation strategies. Finally, four criteria are used to evaluate performance. These are the average within and between dataset prediction correlation, overall RAM usage, and execution time. These values were calculated over the dataset-to-dataset comparisons.

## Supplementary Information

Additional file 1: Supplementary Figures S1–S7. Contains Figures all supplementary figures.

Additional file 2: Table S1. Table containing prediction correlation values for four methods of protein approximation by RNA.

Additional file 3: Table S2. Usability scores for each method, split by publication and performance evaluation.

Additional file 4: Table S3. Prediction correlation values from dataset-to-dataset comparisons as shown in Figure S4.

## Data Availability

Four published CITE-seq datasets were used to evaluate imputation method performance. The first is the Hao et al. PBMC data under accession GSE164378 [11, 46]. The second is non-small cell lung cancer data presented by Leader et al. RNA counts are provided under accession GSE154826 [31, 48] and protein data is available via the github repository [47]. PBMC CITE-seq data from Stephenson et al. is available under the Array Express accession E-MTAB-10026 [32, 49]. The RA synovial data from Zhang et al. is hosted on Synapse under ID syn52297840 [33, 50]. The Nextflow pipeline and accompanying scripts used for method execution and evaluation are available in the associated Github repository under the MIT License [52].
