# Supplementary material for: Machine learning predictions surpass individual mRNAs as a proxy of single-cell protein expression
Source: Genome Biol. 2026 Apr 22;27:185. doi: 10.1186/s13059-026-04083-1 (PMC13235196; doi:10.1186/s13059-026-04083-1)
Supplement: Supplementary file 1 — Additional file 1: Supplementary Figures S1–S7. Contains Figures all supplementary figures. [file 13059_2026_4083_MOESM1_ESM.pdf]

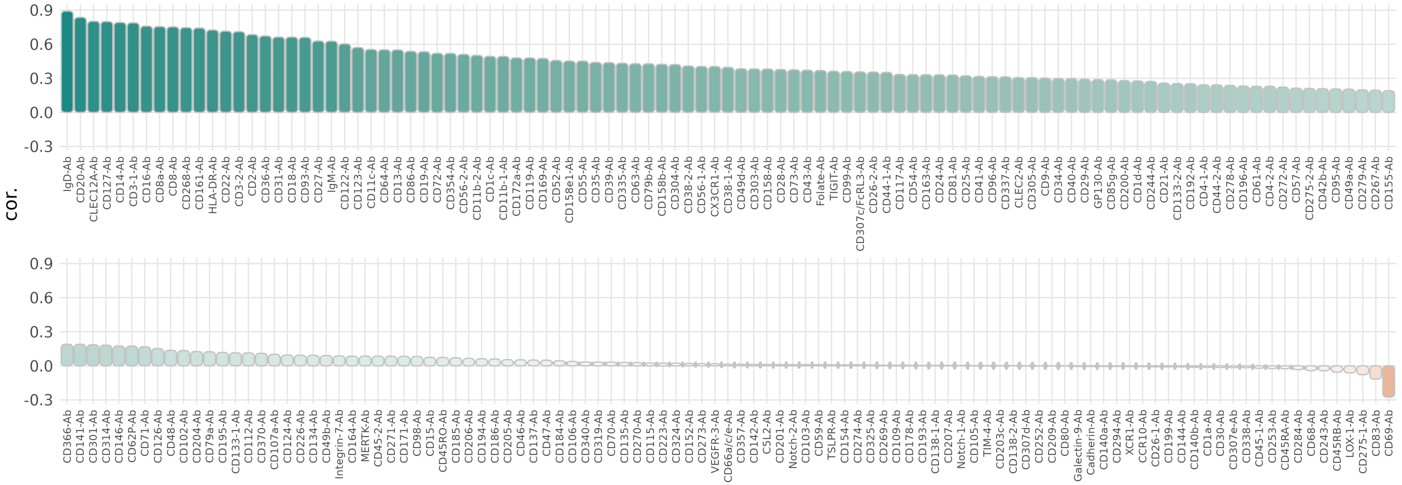

Fig. S1 The correlation between protein and RNA measurements. Bar plot showing Pearson correlation between normalised mRNA and measured extracellular protein. Bar coloured according to value.

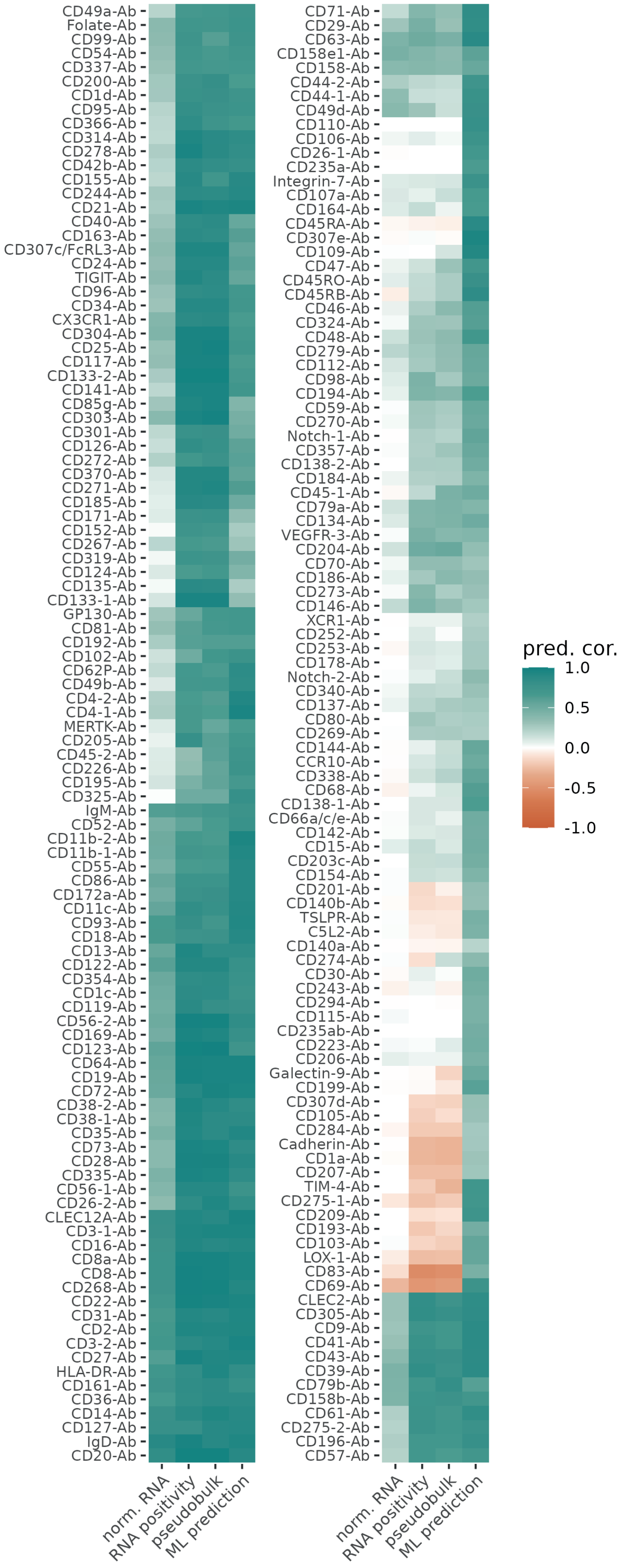

Fig. S2 Comparison of prediction comparison by different approaches. Heatmap of prediction correlation values by estimation approach, with maximum value taken over ML methods.

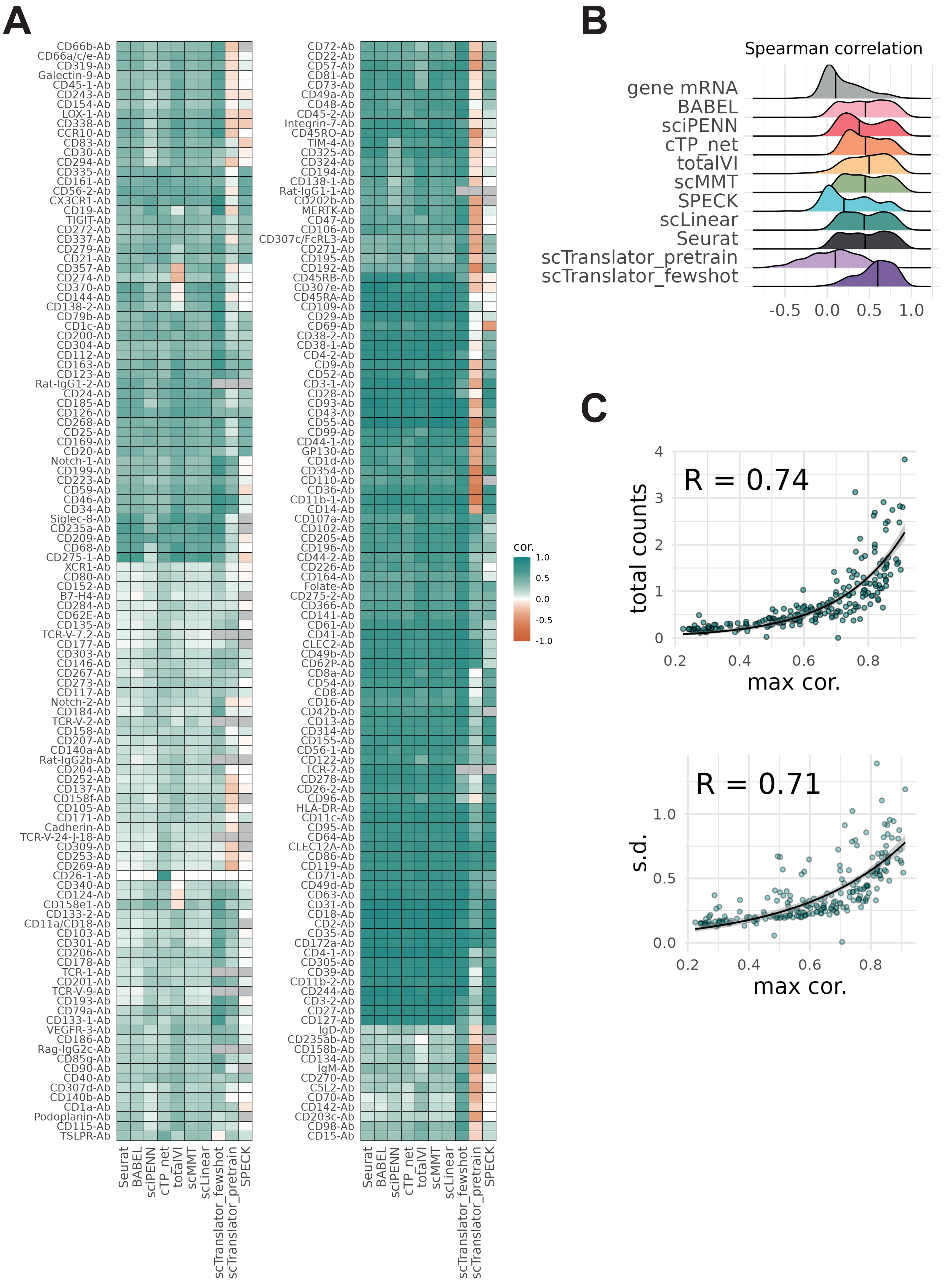

Fig. S3 Spearman prediction correlation outcomes. Spearman prediction correlation results as in Figure 2. A) Heatmap of Spearman prediction correlation for each feature and method. B) Distribution of Spearman prediction correlation over features. C) Scatter plots of total protein count and standard deviation against maximum Spearman prediction correlation.

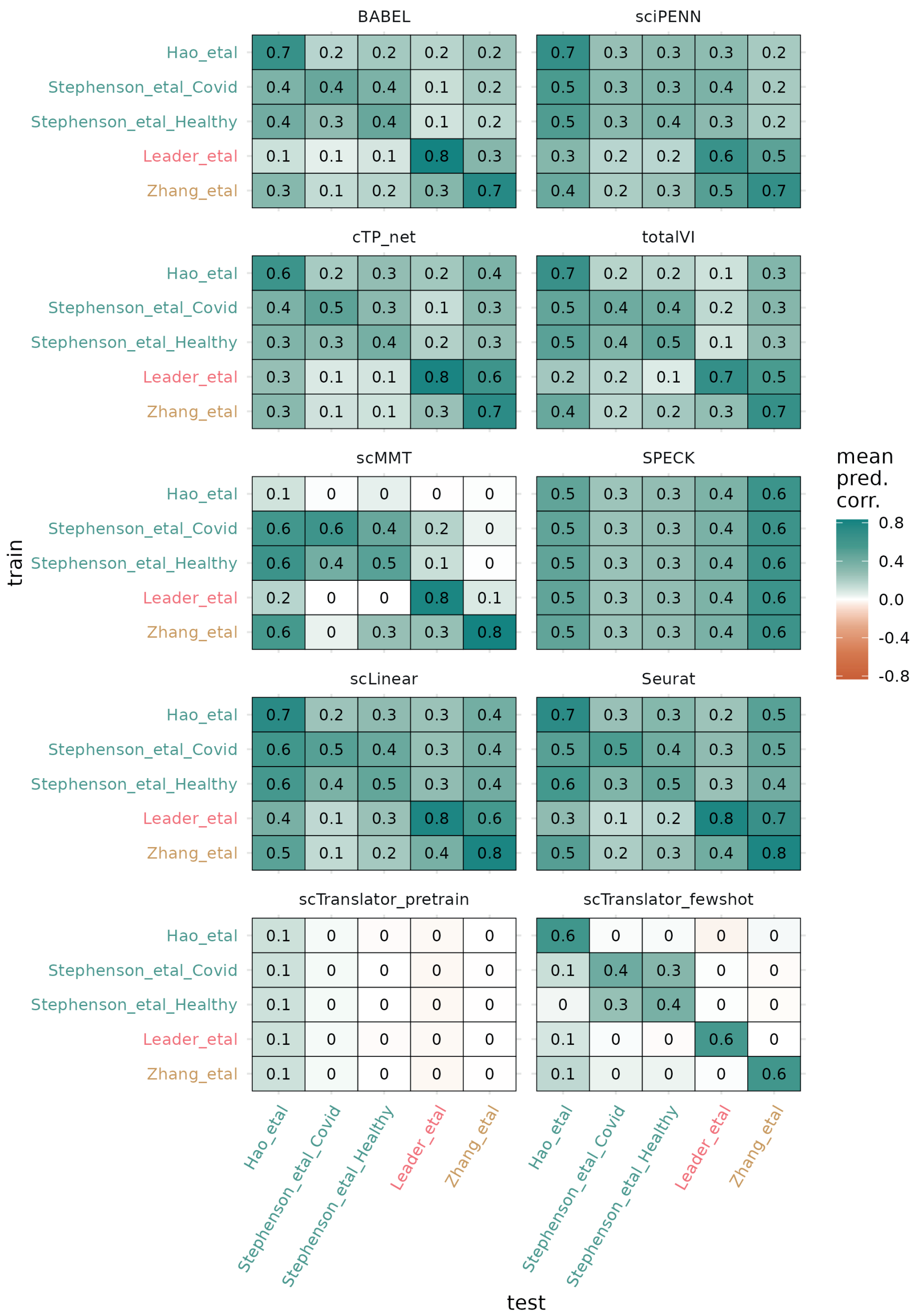

Fig. S4 Prediction correlation in dataset-to-dataset comparison. Heatmap showing median prediction correlation over all antibodies for each method, and for every pairwise combination of training and testing sets in the dataset-to-dataset analysis. Values are rounded to 1 decimal place.

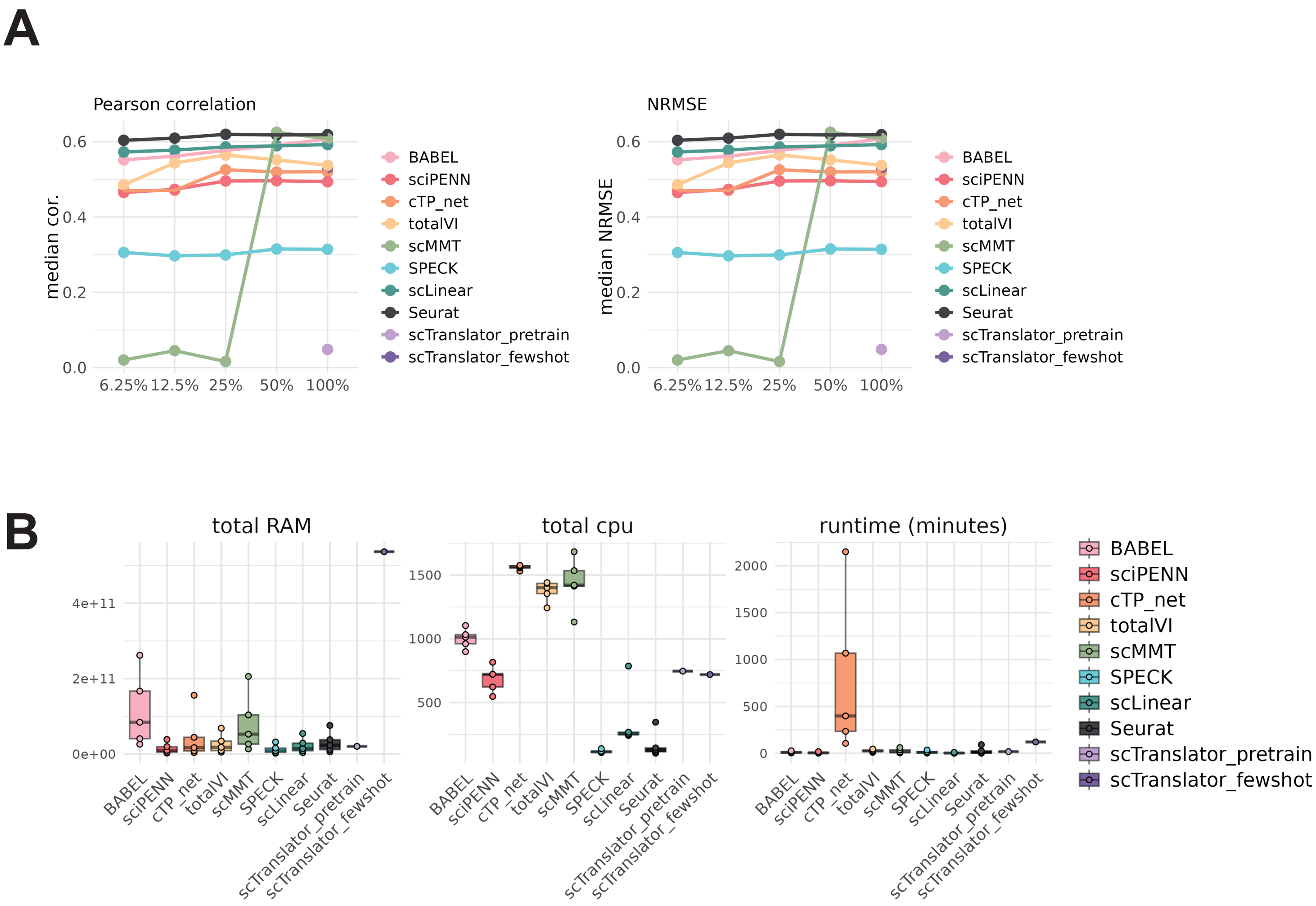

Fig. S5 Performance and resource usage of prediction methods trained on ascending fractions of the Hao et al. data. A) Line plots showing median prediction correlation and NRMSE for each method, as the data size is varied over subsamples. B) Boxplots showing resource usage and runtime for each method, over subsamples of the data. scTranslator shown for 100% fraction only.

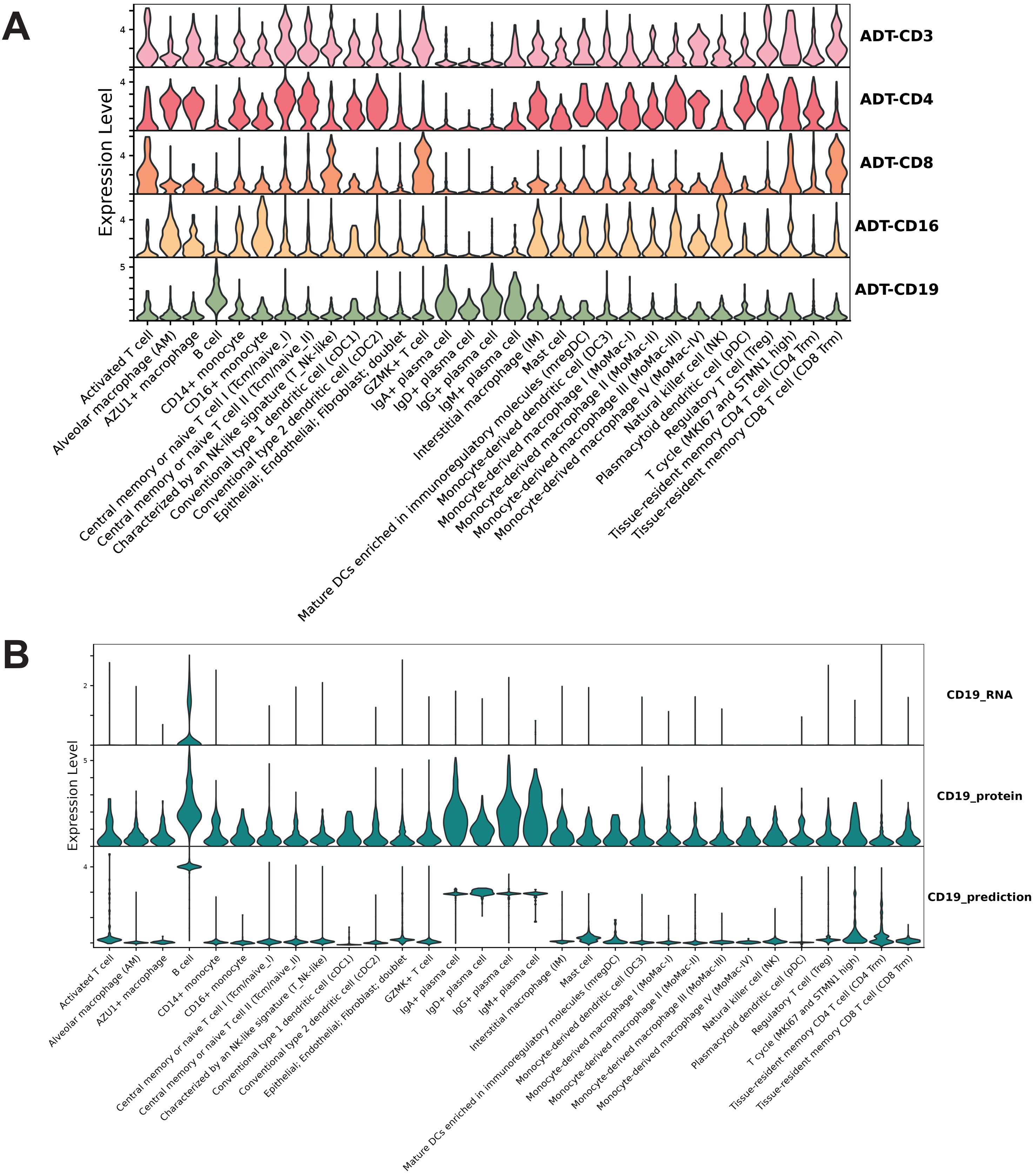

Fig. S6 Recovery of cell type distinction in CD19 expression. A) Normalised expression in the Leader et al. data show for a selection of typical immunophenotyping markers. B) Violins showing expression of CD19 measured by RNA, protein, and Seurat prediction using the Hao et al. data as a training reference.

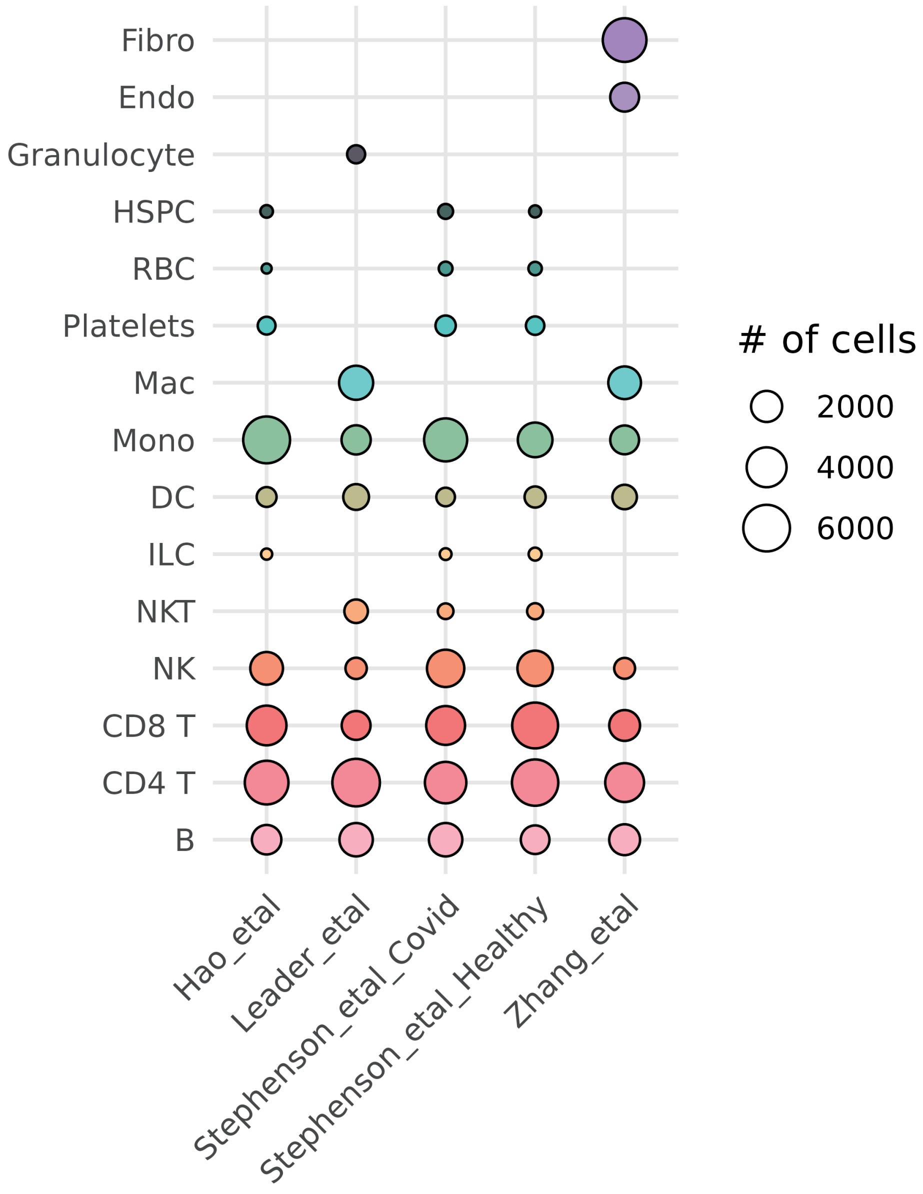

Fig. S7 Composition of training sets. Points with size scaled to represent the number of cells of each type in training sets used for dataset-to-dataset protein imputation.
